# The relationship between Fas and Fas ligand gene polymorphism and preeclampsia risk

**DOI:** 10.1042/BSR20181901

**Published:** 2019-02-15

**Authors:** Tingting Wang, Yunyun Lian

**Affiliations:** Department of Gynaecology and Obstetrics, Affiliated Hangzhou First People’s Hospital, Zhejiang University School of Medicine, Huansha Road, Shangcheng District, Hangzhou, Zhejiang, China

**Keywords:** Fas, FasL, meta-analysis, preeclampsia

## Abstract

Preeclampsia is an idiopathic multisystem disorder with partial genetic and immunological etiology. Several studies investigated the association between various single-nucleotide polymorphisms (SNPs) in Fas and Fas ligand (FasL) genes and the risk of preeclampsia. However, they achieved inconsistent results. Therefore, we conducted a meta-analysis by systematically searching the Cochrane Library, PubMed and Embase databases and assessed this association by calculating pooled odds ratios with 95% confidence interval to reach a more trustworthy conclusion. Subgroup analyses by genotype methods and source of controls (SOC) were also conducted. Seven citations containing nine studies were included for four SNPs (Fas -670 A/G, FasL 124A/G, FasL -844C/T, Fas -1377 G/A) in this meta-analysis. Our data suggested the G allele and genotype GG of the Fas -670 A/G polymorphism, GG genotype of the FasL 124A/G polymorphism, and TT genotype of the FasL -844C/T polymorphism increased the risk of preeclampsia. Stratification analyses by genotype methods and SOC also indicated that Fas -670 A/G polymorphism was related to increased risk for preeclampsia. In conclusion, Fas and FasL gene polymorphisms play important roles in the development of preeclampsia. Further well-designed studies in other races are needed to confirm the findings of this meta-analysis.

## Introduction

Preeclampsia is an idiopathic multisystem disorder with partial genetic and immunological etiology [1]. Preeclampsia is marked by elevatory maternal blood pressure and proteinuria after 20 weeks of pregnancy [2]. There are major geographical differences concerning early onset preeclampsia and late onset preeclampsia throughout the world [3]. The physiopathology remains poorly understood, although the involvement of metabolic, immune, angiogenic, and genetic factors are suggested [3–6]. Several studies [7,8] demonstrated an increased apoptosis level of placental villous trophoblasts in pregnancies complicated by preeclampsia. The Fas Ligand (FasL)–FAS (CD95) system is an essential pathway for the initiation of apoptosis in various cells and tissues [9–11]. Fas and FasL genes, located on chromosomes 10q24.1 and 1q23 respectively, play pivotal roles in the regulation of the apoptotic pathway and immune tolerance in pregnancy and various aspects of mammalian development, especially in immune system homeostasis [12,13]. Therefore, it is reasonable to hypothesize that the Fas and FasL may be candidate genes for preeclampsia susceptibility.

Recently, several studies [14–20] reported the association between single-nucleotide polymorphisms (SNPs) in Fas, FasL genes and the risk of preeclampsia. However, the results were inconsistent and conflicting. For instance, Raguema et al. [14], Salimi et al. [16], Nasr et al. [17], Ciarmel et al. [19], and Sziller et al. [20] all found that Fas -670 A/G polymorphism increased the risk of preeclampsia, whereas Masoumi et al. [15] and Lasabova et al. [18] reported that Fas -670 A/G polymorphism was not related to preeclampsia susceptibility. Therefore, we conducted this meta-analysis to verify whether Fas and FasL gene polymorphisms were associated with preeclampsia risk.

## Materials and methods

### Literature search

We searched the Cochrane Library, PubMed and Embase databases to identify studies through August 30, 2018. The following key words were used: ‘Fas’ or ‘TNFRSF6/’ or ‘CD95’ or ‘APO-1’, ‘Fas Ligand’ or ‘FasL’ or ‘NFSF6’ or ‘CD95L’, ‘SNP’ or ‘polymorphism’ and ‘preeclampsia’ or ‘PE’. No restrictions were placed on the search. Additional initially omitted studies (such as reference lists of identified studies) were identified by hand screening.

### Inclusion and exclusion criteria

The identified studies conformed to the following criteria: (1) studies that evaluated the association between preeclampsia risk and Fas, FasL gene polymorphisms, (2) studies on human beings, (3) studies provided sufficient data to calculate the pooled odds ratios (ORs) and 95% confidence interval (CIs), and *P* value, and (4) case–control studies. Exclusion criteria were as follows: (1) incomplete data; (2) review or case report; (3) duplicate or overlapped publication. All questionable publications were discussed with consensus. Two reviewers independently screened the titles and abstracts.

### Data extraction and quality assessment

Related information was carefully extracted from included studies. The extracted information from all eligible studies including: author name, publication year, nationality, age, sample size, ethnicity, genotype methods, source of controls, and genotype numbers of cases and controls. Two reviewers independently performed the extraction of data and assessed the study quality based on the Newcastle–Ottawa Scale scores (NOS) [21]. Hardy–Weinberg equilibrium (HWE) in controls was tested by Pearson’s χ^2^ test (http://ihg.gsf.de/cgi-bin/hw/hwa1.pl). The NOS criteria were scored according to three aspects: (1) subject selection: 0–4, (2) comparability of subject: 0–2 and (3) exposure: 0–3. The total NOS scores ranged from 0 (lowest) to 9 (highest). All disagreements were discussed and resolved with consensus.

### Statistical analysis

Stata 12.0 software (StataCorp, College Station, TX, U.S.A.) was used to perform all statistical analyses. We assessed the strength of associations between Fas, FasL genes polymorphisms and preeclampsia risk by ORs and 95%CIs. Stratification analyses were carried out by source of controls (SOC) and genotype methods. *P*<0.05 was considered statistically significant. Pooled ORs were calculated for all five gene models (allele, dominant, recessive, homozygous and heterozygous). If a *Q*-test indicated *I*^2^ < 50% or *P*>0.1 indicated heterogeneity across studies, a fixed-effect model was used. Otherwise, the random-effects model was used [22]. We performed sensitivity analyses by leaving out each study in turn to determine the effect on the test of heterogeneity and evaluate the stability of the overall results. Potential publication bias was assessed by both Begger’s and Egger’s linear regression test [23]; *P*<0.05 was considered to indicate statistically significant.

## Results

### Characteristics of the included studies

**Figure 1:**
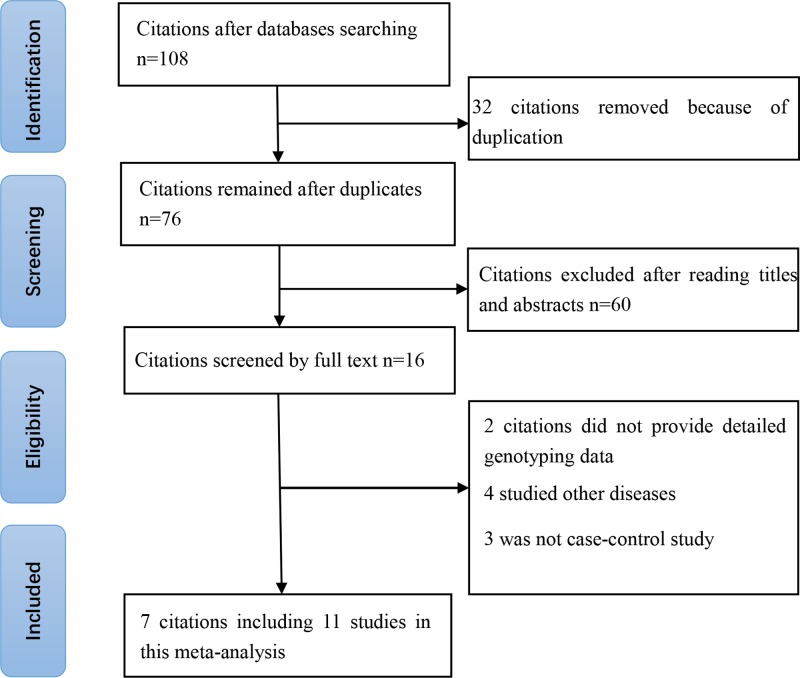
Selection for eligible papers included in this meta-analysis

We yielded a total of 108 citations after incipient search. Sixteen citations were selected for further full-text review. Nine citations were excluded due to the following reasons: two citations did not provide detailed genotyping data; four studied other diseases, and three was not case–control study. Eventually, we identified seven eligible citations [14–20] (834 cases and 1072 controls) containing nine studies. Selection for qualified studies was shown in Figure 1. The characteristics of included studies were summarized in Tables 1 and 2. The NOS of all included studies ranged from 5 to 7 stars, suggesting that these studies were of high quality.

**Table 1:** Characteristics of included studies

Author	Year	Nationality	Sample size		Age (mean)		Study gene	Study SNPs	Genotype method	NOS			HWE
			Case	Control	Case	Control				I	II	III	
Raguema	2018	Tunisia	300	300	30.5	31.3	Fas	-670 A/G	PCR-RFLP	3	1	2	Y
							FasL	124 A/G	PCR-RFLP	3	1	2	Y
Masoumi	2016	Iran	153	140	28.2	27.1	Fas	-670 A/G	PCR-RFLP	4	1	2	Y
								-1377 G/A	PCR-RFLP	4	1	2	Y
							FasL	-844 C/T	PCR-RFLP	3	1	2	N
Salimi	2014	Iran	127	139	28.0	26.6	Fas	-670 A/G	PCR	3	0	2	Y
							FasL	-844 C/T	PCR	3	0	2	N
Nasr	2014	Egypt	50	50	26.3	28.6	Fas	-670 A/G	PCR-RFLP	3	1	2	Y
							FasL	124 A/G	PCR-RFLP	3	1	2	Y
Lasabova (1)	2014	Slovak	46	45	NA	NA	Fas	-670 A/G	PCR	3	0	2	Y
Lasabova (2)	2014	Hungaria	70	78	NA	NA	Fas	-670 A/G	PCR	3	0	2	Y
Ciarmel	2010	Italy	50	142	NA	NA	Fas	-670 A/G	PCR-RFLP	3	0	2	Y
								124 A/G	PCR-RFLP	3	0	2	Y
Sziller (1)	2009	USA	31	89	NA	30.0	Fas	-670 A/G	PCR	3	0	2	Y
Sziller (1)	2009	USA	7	89	NA	30.0	Fas	-670 A/G	PCR	3	0	2	Y

I, Selection; II, Comparability; III, Exposure. Newcastle–Ottawa Scale is available from http://www.ohri.ca/programs/clinical epidemiology/oxford.asp

Abbreviation: RFLP, restriction fragment length polymorphism.

**Table 2:** Genotype distributions of Fas, FasL polymorphisms in the included studies

Author & Year	SOC	Ethnicity	Allele		Case			Control			Association with preeclampsia
			1	2	11	12	22	11	12	22	
**Fas -670 A/G**											
Raguema2018	HB	Caucasians	A	G	105	141	54	151	118	31	Increased risk
Masoumi2016	HB	Caucasians	A	G	58	64	31	47	71	22	Not related
Salimi2014	HB	Caucasians	A	G	27	68	32	64	59	16	Increased risk
Nasr2014	HB	Caucasians	A	G	8	30	12	18	25	7	Increased risk
Lasabova(1)2014	HB	Caucasians	A	G	11	24	11	15	20	10	Not related
Lasabova (1)2014	HB	Caucasians	A	G	14	39	17	23	36	19	Not related
Ciarmel2010	PB	Caucasians	A	G	8	29	13	46	68	28	Increased risk
Sziller2005	HB	Caucasians	A	G	5	15	11	33	37	19	Increased risk
Sziller2005	HB	Caucasians	A	G	2	2	3	33	37	19	Not related
**FasL 124A/G**											
Raguema2018	HB	Caucasians	A	G	99	145	56	152	117	31	Increased risk
Nasr2014	HB	Caucasians	A	G	39	7	4	31	15	4	May decreased risk
Ciarmel2010	PB	Caucasians	A	G	36	12	2	95	38	9	Not related
**FasL -844C/T**											
Masoumi 2016	HB	Caucasians	C	T	58	64	31	70	35	35	Not related
Salimi2014	HB	Caucasians	C	T	22	69	36	30	83	26	Not related
**Fas -1377 G/A**											
Masoumi 2016	HB	Caucasians	G	A	121	28	4	102	38	0	Increased risk

Abbreviations: HB, hospital-based; NA, not available; PB, population-based.

### Meta-analysis of Fas -670 A/G polymorphism

**Figure 2:**
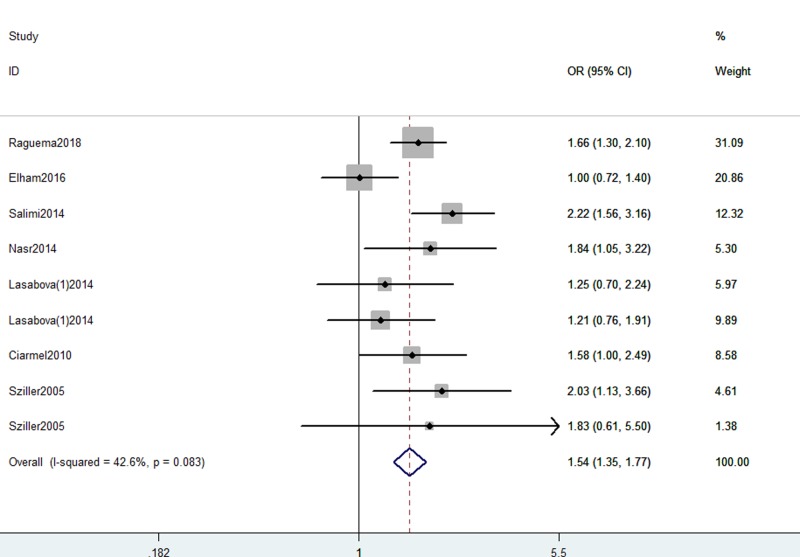
Forest plot shows odds ratio for the association between Fas -670 A/G polymorphism and preeclampsia risk (G vs. A)

**Figure 3:**
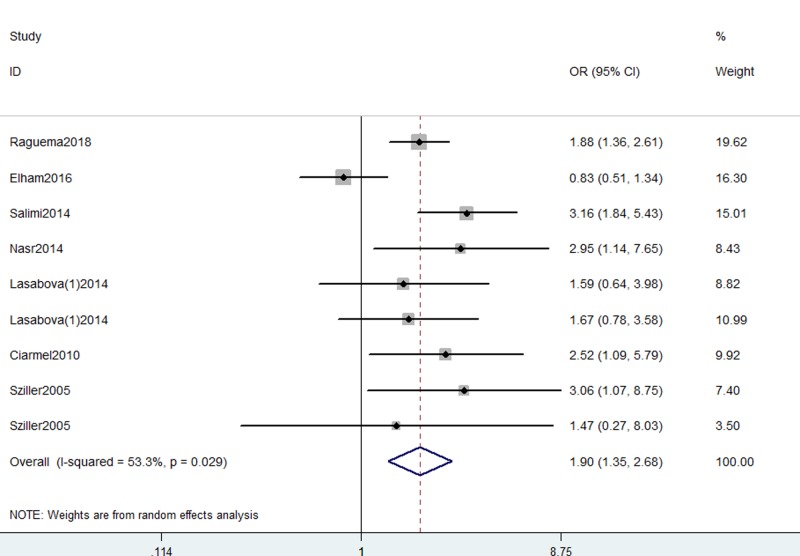
Forest plot shows odds ratio for the association between Fas -670 A/G polymorphism and preeclampsia risk (GG+AG vs. AA)

In the general analysis, we detected a significant association between Fas gene -670 A/G polymorphism with increased risk for preeclampsia (G vs. A: OR, 1.54; 95% CI, 1.35–1.77, *P*<0.001, Figure 2; AG+GG vs. AA: OR, 1.90; 95% CI, 1.35–2.68, *P*=0.029, Figure 3; GG vs. AA+AG: OR, 1.67; 95% CI, 1.31–2.13, *P*<0.001; GG vs. AA: OR, 2.31; 95% CI, 1.75–3.06, *P*<0.001; AG vs. AA: OR, 2.11; 95% CI, 1.34–3.32, *P*=0.001, Table 3). Data indicated that GG genotype and G allele were regarded as risk factors for preeclampsia. Stratification analyses were conducted according to SOC and genotype methods. No different results were found (Table 3).

**Table 3:** Meta-analysis of the association between Fas, FasL gene polymorphisms and preeclampsia risk

SNP	Comparison	Category	Category	Studies	OR (95% CI)	*P*-value	*P* for heterogeneity
Fas -670 A/G	G vs. A	Total (fixed model)		9	**1.54 (1.35, 1.77)**	<0.001	0.083
	Allele model	SOC	HB	8	**1.54 (1.34, 1.77)**	<0.001	0.052
			PB	1	1.58 (0.99, 2.49)	0.051	–
		Genotype method	PCR-RFLP	6	**1.50 (1.28, 1.76)**	<0.001	0.154
			PCR	3	**1.66 (1.29, 2.13)**	<0.001	0.066
	GG+AG	vs. AA	Total (random model)	9	**1.90 (1.35, 2.68)**	<0.001	0.029
	Dominant model	SOC	HB	8	**1.85 (1.27, 2.69)**	0.001	0.021
			PB	1	**2.52 (1.09, 5.79)**	0.030	–
		Genotype method	PCR-RFLP	6	**1.80 (1.13, 2.86)**	0.013	0.030
			PCR	3	**2.25 (1.41, 3.06)**	0.001	0.271
	GG vs. AG+AA	Total (fixed model)		9	**1.67 (1.31, 2.13)**	<0.001	0.673
	Recessive model	SOC	HB	8	**1.70 (1.31, 2.20)**	<0.001	0.588
			PB	1	1.43 (0.67, 3.04)	0.353	–
		Genotype method	PCR-RFLP	6	**1.72 (1.28, 2.30)**	<0.001	0.912
			PCR	3	**1.58 (1.02, 2.43)**	<0.001	0.126
	GG vs. AA	Total (fixed model)		9	**2.31 (1.75, 3.06)**	<0.001	0.216
	Homozygote model	SOC	HB	8	**2.29 (1.71, 3.06)**	<0.001	0.154
			PB	1	2.67 (0.98, 7.24)	0.054	–
		Genotype method	PCR-RFLP	6	**2.20 (1.58, 3.08)**	<0.001	0.335
			PCR	3	**2.60 (1.56, 4.33)**	<0.001	0.092
	AG vs. AA	Total (random model)		9	**2.11 (1.34, 3.32)**	0.001	0.001
	Heterozygote model	SOC	HB	8	**1.82 (1.19, 2.79)**	0.006	0.010
			PB	1	**5.75 (2.31, 14.29)**	<0.001	–
		Genotype method	PCR-RFLP	6	**2.53 (1.25, 5.11)**	0.009	<0.001
			PCR	3	**1.94 (1.28, 2.93)**	0.002	0.557
FasL 124A/G	G vs. A	Total (random model)		3	0.99 (0.47, 2.07)	0.968	0.002
	Allele model	SOC	HB	2	1.08 (0.37, 3.13)	0.890	0.005
			PB	1	0.78 (0.42, 1.43)	0.413	–
	GG+AG	vs. AA	Total (random model)		0.97 (0.38, 2.51)	0.951	0.001
	Dominant model		HB	2	1.04 (0.24, 4.54)	0.961	0.002
			PB	1	0.79 (0.39, 1.60)	0.506	–
	GG vs. AG+AA	Total (fixed model)			**1.70 (1.11, 2.59)**	0.014	0.277
	Recessive model		HB	2	**1.87 (1.19, 2.92)**	0.006	0.0374
			PB	1	0.62 (0.13, 2.95)	0.544	–
	GG vs. AA	Total (random model)			1.34 (0.45, 3.98)	0.603	0.069
	Homozygote model		HB	2	1.81 (0.57, 5.78)	0.318	0.114
			PB	1	0.59 (0.12, 2.85)	0.508	–
	AG vs. AA	Total (random model)			0.92(0.36, 2.35)	0.860	0.003
	Heterozygote model		HB	2	0.90(0.18, 4.46)	0.900	0.003
			PB	1	0.83(0.39, 1.77)	0.636	–
FasL -844C/T	T vs. C	Total (fixed model)			1.24 (0.98, 1.57)	0.077	0.608
	Allele model						
	TT+TC vs. CC	Total (fixed model)			**1.51 (1.04, 2.19)**	0.029	0.574
	Dominant model						
	TT vs. TC+CC	Total (random model)			1.14 (0.51, 2.53)	0.748	0.045
	Recessive model						
	TT vs. CC	Total (fixed model)			1.33 (0.84, 2.12)	0.222	0.243
	Homozygote model						
	TC vs. CC	Total (random model)			1.62(0.84, 3.10)	0.148	0.117
	Heterozygote model						

*Bold values are statistically significant (*P*<0.05).

We assessed sensitivity analysis by leaving out each study in turn in each genetic model for -670 A/G polymorphism. The pooled ORs for the effects of the SNPs on the risk for preeclampsia risk indicated that our data were credible. Both Egger’s and Begg’s tests were used to evaluate the publication bias of this meta-analysis. Our data revealed that there was no obvious publication bias for Fas -670 A/G polymorphism (data not shown).

### Meta-analysis of FasL gene 124A/G and -844C/T polymorphisms

Results of pooled analysis on the association between FasL gene 124A/G polymorphism and preeclampsia risk were shown in Table 3. GG genotype (GG vs. AA+AG: OR, 1.70, 95% CI, 1.11–2.59, *P*=0.014) for the 124A/G polymorphism increased the risk of preeclampsia. We also detected a significant association between FasL gene -844C/T polymorphism with increased risk for preeclampsia (TT+TC vs. CC: OR, 1.51, 95% CI, 1.04–2.19, *P*=0.029).

The Fas gene -1377 G/A polymorphism was investigated only in one study [15], which reported a significant association (Table 2). Nevertheless, further replication studies are required to confirm the associations.

## Discussion

In this meta-analysis, our data showed that the Fas -670 A/G polymorphism, FasL 124A/G polymorphism, and FasL -844C/T polymorphism increased the risk of preeclampsia among Caucasians. Stratification analyses of genotype methods and SOC also yielded similar increased risks for preeclampsia.

Some studies provided evidence that disturbances in apoptosis were associated with increased risk for preeclampsia [1,19]. Fas and FasL pathways are involved in the regulation of immune tolerance in pregnancy, apoptotic pathways, and various aspects of mammalian development [12,24]. Recently, many studies investigated the association between Fas and FasL gene polymorphisms and preeclampsia risk. However, they obtained inconsistent findings. Sziller et al. [20] first reported the Fas A-670G polymorphism in 38 pregnant women with preeclampsia and 89 controls. They showed that Fas A-670G polymorphism was associated with increased risk for preeclampsia in overall analysis [20]. In addition, subgroup analysis also indicated that this SNP was related to risk of preeclampsia-associated intrauterine growth restriction in women who deliver at <37 weeks [20]. Similar increased risks were replicated in an Italian population study by Ciarmela et al. [19], an Iranian population by Salimi et al. [16], an Egyptian population by Nasr et al. [17], and a Tunisian population by Raguem et al. [14]. It is of note that the finding of Masoumi et al. [15] from southeast Iran was in contrast with the investigation by abovementioned studies. Ethnicity factor cannot explain the contradictory results of Salimi et al. [16] and Masoumi et al. [15], because they were both from the Iranian population. Study with 116 preeclamptic women and 123 healthy control subjects from Lasabova et al. [18] also did not obtain positive findings for Fas A-670G polymorphism. Due to the conflicting results of these studies, it is necessary to conduct a meta-analysis to address these issues. Meta-analysis is utilized to combine the data based on a single study to yield conclusive conclusions. In this meta-analysis, we found that Fas -670 A/G polymorphism increased the risk of preeclampsia among Caucasians. Stratification analyses of genotype methods and SOC also uncovered similar results for preeclampsia. As for the remaining three SNPs, this meta-analysis suggested that the FasL 124A/G and FasL -844C/T polymorphisms also were related to increased risk for preeclampsia. Only one study [15] explored Fas -1377 G/A polymorphism and showed this SNP increased the risk of preeclampsia. To our best knowledge, this study is the first meta-analysis investigating the association between Fas and FasL gene polymorphisms and preeclampsia susceptibility.

Some limitations in this meta-analysis should be considered. First, the heterogeneity of this meta-analysis was somewhat high. Second, we could not conduct some stratification analyses of other potential factors including smoking and drinking. Third, our results were based on unadjusted estimates for confounding factors, which might have affected the final results. Fourth, because of the lack of relevant data, potential gene–gene and gene–environment interactions were not performed. Fifth, the sample sizes of this meta-analysis were not large, which may lead to reduced statistical power. Last but not least, we did not explore the Asian populations due to lack of relevant studies.

In conclusion, this meta-analysis indicates that Fas and FasL gene polymorphisms increase the risk of preeclampsia risk. Further studies with large sample sizes in other populations are urgently needed to confirm the findings of this meta-analysis.

## Abbreviations

CI: confidence interval
FasL: Fas ligand
HWE: Hardy–Weinberg equilibrium
NOS: Newcastle–Ottawa Scale scores
OR: odds ratio
RFLP: restriction fragment length polymorphism
SNP: single-nucleotide polymorphism
SOC: source of controls
